# Transcriptomics: A Step behind the Comprehension of the Polygenic Influence on Oxidative Stress, Immune Deregulation, and Mitochondrial Dysfunction in Chronic Kidney Disease

**DOI:** 10.1155/2016/9290857

**Published:** 2016-06-21

**Authors:** Simona Granata, Alessandra Dalla Gassa, Gloria Bellin, Antonio Lupo, Gianluigi Zaza

**Affiliations:** Renal Unit, Department of Medicine, University Hospital of Verona, Piazzale Stefani 1, 37126 Verona, Italy

## Abstract

Chronic kidney disease (CKD) is an increasing and global health problem with a great economic burden for healthcare system. Therefore to slow down the progression of this condition is a main objective in nephrology. It has been extensively reported that microinflammation, immune system deregulation, and oxidative stress contribute to CKD progression. Additionally, dialysis worsens this clinical condition because of the contact of blood with bioincompatible dialytic devices. Numerous studies have shown the close link between immune system impairment and CKD but most have been performed using classical biomolecular strategies. These methodologies are limited in their ability to discover new elements and enable measuring the simultaneous influence of multiple factors. The “omics” techniques could overcome these gaps. For example, transcriptomics has revealed that mitochondria and inflammasome have a role in pathogenesis of CKD and are pivotal elements in the cellular alterations leading to systemic complications. We believe that a larger employment of this technique, together with other “omics” methodologies, could help clinicians to obtain new pathogenetic insights, novel diagnostic biomarkers, and therapeutic targets. Finally, transcriptomics could allow clinicians to personalize therapeutic strategies according to individual genetic background (nutrigenomic and pharmacogenomic). In this review, we analyzed the available transcriptomic studies involving CKD patients.

## 1. Introduction

Chronic kidney disease (CKD) represents an increasing global worldwide health problem particularly in elderly people [1–3] and/or affected by diabetes, hypertension, and obesity [4–6]. Therefore, understanding the biological machinery associated with CKD represents an important objective in nephrology and internal medicine.

During this condition, patients experience a gradual loss of renal function over time with a progressive decline in the glomerular filtration rate (GFR). An international consensus categorized CKD into 5 stages according to the GFR [7]. In the last stage of renal failure (called end stage renal disease, ESRD) biochemical changes are incompatible with life and renal replacement therapies (RRT: hemodialysis (HD) and peritoneal dialysis (PD)) are necessary [8–11].

As the kidney is a complex and highly specialized organ, with different functions (e.g., pH, plasma and tissue hydrosaline balance, and vitamin D and erythropoietin production), chronic kidney impairment may also determine significant metabolic and endocrine changes (including acidosis, hyperparathyroidism, and anemia) that may induce relevant clinical complications (e.g., atherosclerosis, pericarditis, osteodystrophy, and uremic encephalopathy) [12, 13].

Consequently, a healthy life style, with an adequate physical activity and an equilibrate diet, with low salt intake and no smoking habit, can undeniably prevent or slow down the progression of renal damage and lessen complications [14–17]. Treating diabetes and hypertension is also mandatory to control CKD and, as largely described, the introduction of angiotensin-converting enzyme (ACE) inhibitors and angiotensin receptor blockers (ARBs) is recommended to potentiate nephroprotection [18–23]. Low protein diets, then, are required to reduce uremic symptoms until ESRD [24].

Additionally, the removal of the oxidative stress and microinflammatory insults represents an additional powerful therapeutic target in CKD [25, 26]. Unfortunately, in most cases, this approach results in being ineffective and not capable of stopping the progression of the kidney damage toward renal failure. Moreover, during dialysis, the contact of blood and/or tissues with bioincompatible devices may dramatically worsen this pathological status [27–29].

## 2. Immune-Inflammatory Deregulation in CKD

**Figure 1:**
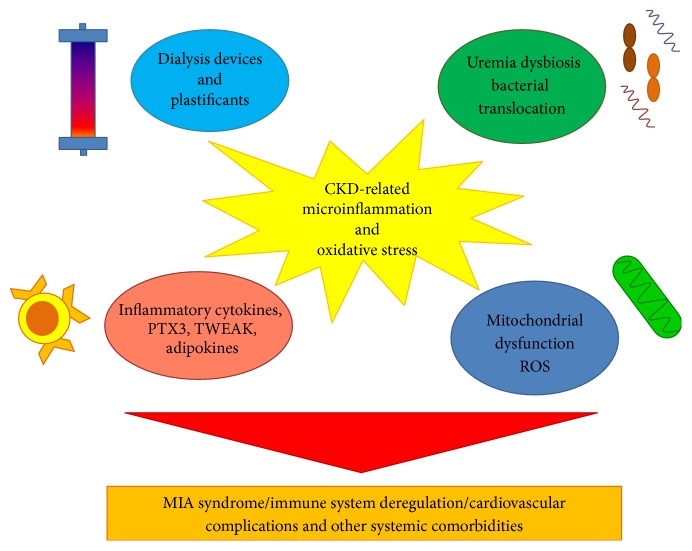
Schematic representation of the main factors involved in microinflammation and oxidative stress in chronic kidney disease (CKD). As reported, (1) bioincompatible dialysis devices and plastificants; (2) classical inflammatory cytokines and new emerging biological elements such as pentraxin-3 (PTX3), TNF-like weak inducer of apoptosis (TWEAK), and adipokines; (3) uremia-induced intestinal dysbiosis with an increased translocation of gut bacteria and bacterial components into the circulation; and (4) mitochondrial deregulation may have a central role in the onset of chronic microinflammatory state and oxidative stress and development of malnutrition, inflammation, and atherosclerosis (MIA) syndrome, systemic complications, immune system deregulation, cardiovascular complications, and other systemic comorbidities in CKD patients.

Several factors may be responsible of the chronic immune-inflammatory state and oxidative stress in CKD patients (Figure 1) and classical inflammatory cytokines (tumor necrosis factor-*α* (TNF-*α*), interleukin- (IL-) 1, IL-6, and IL-10) [30–32] and new emerging biological elements such as pentraxin-3 (PTX3) [33, 34] and TNF-like weak inducer of apoptosis (TWEAK) seem involved and significantly correlated to the degree of the renal damage [35–37].

PTX3, a circulating acute phase protein with pattern recognition molecule properties and with antibody-like functions, contributes to innate immunity defence against pathogens and in the regulation of inflammation in CKD [35–37].

TWEAK can increase secretion of other cytokines locally in the kidney and its blood levels seem independently associated with coronary artery disease in patients with renal damage [38, 39].

Adipokines, together with visfatin and leptin, were found higher in CKD and in nondiabetic peritoneal dialysis patients. Interestingly, leptin/adiponectin ratio was able to predict mortality in a group of nondiabetic uremic patients undergoing peritoneal dialysis treatment [40–42].

Interestingly, convincing recent evidences suggest that uremia-induced intestinal dysbiosis may have a central role in these processes by increasing the translocation of gut bacteria and bacterial components into the circulation, which can in turn activate systemic inflammation [43–45].

A recent cross-sectional study in stage 3-4 CKD demonstrated that indoxyl sulfate and p-cresyl sulfate (nephro- and cardiovascular toxins produced solely by the gut microbiota) were associated with elevated levels of inflammatory biomarkers as well as with increased arterial stiffness [46].

Additionally, it has been largely reported that CKD patients develop a complex immune dysfunction with an interaction between the innate and adaptive systems, in which immune activation (hypercytokinemia and acute phase response) and immune suppression (impairment of response to infections and poor development of adaptive immunity) coexist [47]. Moreover renal replacement therapies worsen the immune system deregulation.

CKD progression and dialysis procedure contribute also to protein-energy malnutrition, probably mediated by proinflammatory cytokines that can affect appetite and increased protein hydrolysis and muscle protein breakdown [48]. The association between malnutrition, inflammation, and atherosclerosis in this patient population has suggested the existence of a syndrome called malnutrition, inflammation, and atherosclerosis (MIA), which is associated with an exceptionally high mortality rate [49].

To minimize these dialysis-related conditions [50, 51] several pharmacological industries and researchers are working closely. However, at present, despite the great efforts, we are still far from the standardization of a full biocompatible dialysis procedure.

## 3. Oxidative Stress and Mitochondrial Dysfunction in CKD

Oxidative stress and mitochondrial deregulation play a major role in CKD and, already from the early stage of CKD, several markers of oxidative stress (e.g., malondialdehyde, F2 isoprostanes, and advanced oxidation protein products) are plentiful with a concomitant decrease of antioxidants (e.g., superoxide dismutase, glutathione peroxidase, and vitamins E and C) [26, 52–55].

The accumulation of uremic toxins, through the direct augmentation of NADPH oxidase and xanthine oxidoreductase activities [56, 57], the inflammasome activation, and the additional prooxidative stimuli due to the contact of PBMCs with dialytic devices may induce a significant increment of reactive oxygen species (ROS) [58, 59]. ROS are active molecules able to oxidize proteins, lipids, and nucleic acids with a subsequent damage of cells and tissues [26, 60].

Likewise, during PD conventional dialysis solutions, containing high concentrations of glucose and glucose degradation products, may increase ROS production in human peritoneal mesothelial cells with consequent loss of ultrafiltration capacity, increased vascular density, and development of fibrosis [61].

Oxidative stress is also accountable for the onset and development of severe clinical complications (including cardiovascular disease, atherosclerosis, hypertension, anemia, and malnutrition) with a consequent low quality of life, high risk of hospitalization, and short survival of CKD patients in both conservative and dialysis treatment [28, 62–64].

Notably, recent studies have suggested that mitochondria could be implicated in this CKD-associated prooxidative machinery [9, 65]. These organelles are involved in numerous functions: ATP synthesis by oxidative phosphorylation, fatty acids *β*-oxidation, synthesis of heme, apoptosis, synthesis of steroid hormones, nitrogen balance through urea cycle, and Ca homeostasis [66–68].

Structurally, they present an outer and inner membrane, the latter of which would be impermeable to all molecules in the absence of specific carriers and contains the OXPHOS complexes. Electrons derived from metabolic reducing equivalents (NADH and FADH_2_) enter into the electron transport chain through either complex I or complex II and via respiratory chain to molecular oxygen which is finally reduced to water. This exergonic process is used to pump protons from the mitochondrial matrix into the intermembrane space, creating an electrochemical gradient used by complex V for ATP synthesis [69].

During this process a small percentage (0.4–4%) of electrons may “leak” from the respiratory chain (in particular at complexes I and III) and partially reduce oxygen, forming superoxide anion (O_2_^−^) [70]. Consequently mitochondria are the major source of ROS in the cell.

Additionally, during CKD, patients' cells undergo reduction in mitochondrial DNA (mtDNA) copy number, loss of mitochondrial membrane potential (Δ*ψ*_m_), and drop of ATP production [71].

Mitochondria are also involved in apoptosis and epithelial to mesenchymal transition of renal tubular epithelial cells contributing to the fibrogenic process [72].

## 4. Transcriptomic Analysis Revealed an Unrecognized and Specific Proinflammatory and Prooxidative Biological/Cellular Machinery in CKD

In the last two decades, researchers have tried to identify key regulators of the intricate inflammatory pathway activated by uremia “per se” and by dialysis, but, most of the time, this research strategy based on single factor analysis is limited and biased. Hence, a simultaneous multifactorial analysis in CKD appears more powerful and effective.

The recent development and extension of high-throughput technologies have allowed reaching the above-mentioned objective. In particular, microarray technology, which allows the study of the entire transcriptome thanks to the hybridization of nucleic acid (RNA) with dozens of thousands of DNA probes attached to a solid support (such as glass, plastic or silicon), has been revealed to be promising. Briefly, transcripts extracted from samples are labeled with fluorescent dyes and hybridize to their complementary targets. Light intensity is then an indirect measurement of gene expression. Transcriptome is the sum of RNA transcripts that comprehend messenger RNAs, ribosomal and transfer RNA, and regulatory noncoding RNAs [81–85].

This technology produces a large amount of raw data that require specific statistical and bioinformatics tools in order to avoid or minimize false positive and to obtain “more conservative” results. In this context, a well conducted validation process by using standardized classical biomolecular methodologies can reduce these biases.

As the other high-throughput (omics) sciences, no prior hypothesis is made. Relationships among top selected genes are, then, translated into biological pathway by using specific software for functional analysis (e.g., Ingenuity Pathway Analysis) [31, 86].

In the last ten years numerous studies have used this approach in nephrology and the results have been very useful in discovering new insights in the pathogenesis of CKD as well as in the comorbidities associated with renal failure (Table 1).

Our group, in the 2008, has published one of the first studies using this technology in dialysis [10]. Results of this study clearly demonstrated, for the first time in dialysis, that a group of genes (e.g.,* MIF*,* IL8RB*, and* CXCL12*) has a causative role in the microinflammatory/oxidative stress in HD and PD patients and they were able to discriminate dialysis from undialyzed CKD subjects (in pharmacological conservative therapy). C-X-C motif chemokine 12 (CXCL12) and IL-8 receptor, beta (IL8RB) were inversely correlated to C-reactive protein (CRP) levels and highly expressed in PD and CKD patients, respectively. Macrophage migration inhibitory factor (MIF) was upregulated in HD patients and directly correlated to CRP levels.

CXCL12 and its receptor, CXCR4, are important modulators of inflammation and immune response. IL8RB binds IL-8, a chemokine with proinflammatory and chemotactic activity [87]. Previous reports have shown a decreased surface expression of this receptor on PBMC of patients with severe chronic inflammatory disease [88, 89]. MIF encodes an “early response” cytokine that plays an important pathogenic role in numerous inflammatory disorders [90, 91]. It activates macrophages to produce proinflammatory mediators and to migrate to the sites of inflammation [92, 93]. In a mouse model of spontaneous atherosclerosis, MIF blockade led to a marked reduction of inflammation associated with the disease [94].

These data were in line with those obtained by Friedrich et al. [73] that, using a combined microarray and RT-PCR approach, demonstrated that HD treatment significantly increased the transcript levels of several proinflammatory cytokines, such as TNF-*α* and IL-8, C-C chemokine receptor type 7 (CCR7), and the CX3C chemokine receptor 1 (CX3CR1). Dividing the patients into two groups, “high CRP levels” and “low CRP levels,” they found that in the latter the increment of transcript levels of anti-inflammatory cytokine receptors (IL-1RN, IL-4R, IL-10R, and IFN*γ*-R1) and chemokine receptors (CX3CR1, CXCR4, and CCR7) was significantly more pronounced than in the high CRP group.

Similar results were obtained by Shah et al. [74] that compared gene expression profile in muscle of healthy subjects versus HD patients and its dynamic change in response to HD. Genes in response to HD were a dynamic response to activation of inflammation, apoptosis, and alterations in cell cycle. In particular, GADD45B, a TNF*α*-inducible gene and a physiological target of nuclear factor-*κ*B (NF-*κ*B), was upregulated [95]. The protective activity of GADD45B against TNF-*α*-induced programmed cell death involves suppression of the c-Jun-N-terminal kinase cascade, a pathway usually associated with cell death, and this suppression is central to control of apoptosis by NF-*κ*B.

Transcriptomic profile was also influenced by different dialysis procedures. Wilflingseder et al. clearly demonstrated with microarray analysis conducted in PBMCs of four stable HD patients that a large group of genes (*n*: 172) were upregulated after treatment with semisynthetic membranes when compared to full-synthetic membranes [75]. These genes were involved in immunity and defence, signal transduction, and apoptosis. Dialysis with a full-synthetic membrane, on the other hand, led to activation of 72 genes that were mainly involved in cell cycle and cell cycle control.

Surprisingly, these results were different from those published by Hochegger et al. [76] that did not find any significant genetic difference between human polymorphonuclear neutrophils (PMN) stimulated with cuprophane versus polysulfone. When these results were combined to one group, the comparison with unstimulated cells revealed 50 genes differentially expressed with a marked upregulation of FOS- and JUN-transcripts, but with only little activation of immune response genes, and, contrarily to PMN stimulated with E. Coli, no upregulation of apoptosis related transcripts.

Subsequently, our research group performed a research project aimed at understanding the influence of dialysis on the PBMCs' immunotranscriptome [29]. As result, we showed that patients with advanced renal impairment in conservative pharmacological treatment (CKD stages III-IV) exhibited a large amount of differentially expressed mRNAs compared to those undergoing HD.

Among the genes downregulated in HD patients, we identified those encoding the human leukocyte antigen- (HLA-) G, a nonclassical major histocompatibility complex class I molecule that differs from other HLA class I molecules with regard to its low polymorphism, restricted tissue distribution, slow turnover, immunosuppressive properties, and limited peptide diversity [96–98].

Under physiological conditions, the production of HLA-G protein is restricted to trophoblast [99], thymic epithelial cells [100], first-trimester placental chorionic blood vessel endothelial cells [101], and IFN-*γ*-treated mononuclear phagocytes [102]. However, the upregulation of this protein can be detected in several pathological conditions such as transplantation, tumors, viral infections, and autoimmune diseases [103–107].

HLA-G possesses the capability to bind inhibitory receptors such as the immunoglobulin-like transcripts 2 and 4 (ILT2, ILT4) and the killer immunoglobulin-like receptor (KIR)2DL4/CD158d with inhibitory effects [107, 108].

HLA-G may also have a direct immune-inhibitory function through blocking effector cells and indirect immune-inhibitory activity by regulatory cell generation. Via the direct inhibitory functions, HLA-G is able to inhibit the cytolytic activity and proliferation of NK [109], the antigen-specific cytolytic functions of *α*/*β* and *γ*/*δ* T lymphocytes [109, 110], the alloproliferative response of T cells [111, 112], the proliferation of NK and T cells [113], and the DCs maturation [114].

Therefore, it is plausible that the lower HLA-G expression in HD patients may determine a hyperactivation of T cells and NK that could explain the different immune response of dialyzed patients to viral infections and tumors.

Recently, Scherer et al. [77], in a study conducted in a large subset of patients using Affymetrix Human Genome U133 Plus 2.0 arrays, confirmed an important immune-transcriptomic deregulation in CKD/ESRD patients and they reported that several genes involved in complement pathway and oxidative metabolism and in response to stress and injury were upregulated in uremia, while transcripts associated with the clathrin-coated vesicle endosomal pathway were markedly reduced consistent with a defect in phagocytosis. Key genes in the immune synapse and the T-cell receptor signaling pathway were reduced, including MHC-class II and the T-cell receptor alpha/beta heterodimer, the coassociated CD3 and CD4 molecules, and a variety of downstream signaling components of the T-cell receptor pathway, the CD28 receptor pathway, and the IL-2 response and signaling pathway.

Moreover, Yokoi et al. [78] in a very well conducted and elegant microarray study demonstrated an upregulation of pleiotrophin in chlorhexidine gluconate (CG) induced peritoneal fibrosis mice versus controls.

This growth factor was found not only in fibroblasts and mesothelial cells within the underlying submesothelial compact zones of mice, but also in human peritoneal biopsy samples and peritoneal dialysate effluent. In wild-type mice, CG treatment increased peritoneal permeability, increased mRNA level of TGF-*β*1, TNF-*α*, and IL-1*β*, resulted in infiltration of CD3-positive T cells, and caused a high number of Ki-67-positive proliferating cells. Authors concluded that the upregulation of pleiotrophin could play a central role in local/systemic inflammation and fibrosis during peritoneal injury.

Also genes involved in proteoglycans biosynthesis/metabolism appear differentially expressed in dialyzed patients [79] compared to healthy subjects (HS) or subjects with a low degree of renal impairment (CKD/HS). Twenty-five genes were upregulated (e.g.,* HPSE*,* VCAN*, and* VEGFA*) and 45 downregulated (e.g.,* IDS* and* HEXA*) in PD/HD compared to CKD/HS. As well, gene expression and plasma activity of heparanase (HPSE), one of the top selected upregulated genes in PD/HD, were significantly correlated with the inflammatory state measured by high-sensitive C reactive protein (HS-CRP).

These results demonstrated that PBMCs of uremic patients undergoing both peritoneal and hemodialysis exhibit a chronic activation of the biosynthetic proteoglycans transcriptomic pattern with heparanase being a central biological element. This enzyme could be considered important for rolling and leukocytes mobility in response of pathological dialysis stimuli. In future, a pharmacological modulation of HPSE could definitely mitigate these effects and reduce the frequent CKD-associated vascular comorbidities.

Transcriptomic analysis demonstrated different expression of several genes involved in oxidative phosphorylation system (OXPHOS) and mitochondrial function in ESRD/HD patients compared to healthy subjects [9]. In particular, complex IV activity, the terminal enzyme of the mitochondrial respiratory chain that transfers the electrons from reduced cytochrome c to oxygen [115], was significantly lower in CKD/HD patients compared to healthy subjects demonstrating a reduced activity of OXPHOS in this population. This is an important finding because it clearly demonstrated that this organelle has a central role in the biological machinery associated with renal failure.

Mitochondria are major source of cellular ATP molecules, but if damaged, they may generate high levels of ROS with massive clinical systemic consequences. Therefore, modulating their function and biogenesis could turn to be a valuable therapeutic option.

Finally, a combined research strategy between classical biomolecular strategies and high-throughput techniques showed the Nod-like receptor protein 3 (NLRP3) inflammasome activation in dialyzed CKD patients [80]. NLRP3-inflammasome is a cytoplasmic multiprotein complex of three proteins: (1) NLRP3, (2) apoptosis-associated speck-like protein containing CARD domain (ASC), and (3) caspase 1 (CASP-1). This complex is involved in the immune response by activating two proinflammatory cytokines: IL-1*β* and IL-18 [116].

NLRP3 inflammasome can be activated by a lot of exogenous and endogenous stimuli: pathogen-associated molecular patterns (PAMPs), such as bacterial and viral RNA [117–119], and damage-associated molecular patterns (DAMPs) such as urate crystals, calcium oxalate crystals, high glucose, extracellular ATP, oxidized mitochondrial DNA, and ROS [120].

The activation of NLRP3 inflammasome requires 2 specific signals. The first, or priming signal, converges on NF-*κ*B to induce the transcription of inflammasome components, IL-1*β* and IL-18, and the second signal leads to inflammasome assembly [116].

ROS being able to activate the proinflammatory transcription factors [121, 122] have a key role in the priming of inflammasome activation [123].

The central role of mitochondrial ROS and NLRP3 activation has been reported also in the pathogenesis of albumin-induced renal tubular injury [124–126].

## 5. Transcriptomics May Facilitate the Development of a Personalized Medicine for the Treatment of Microinflammation and Oxidative Stress in CKD: Looking to the Future

**Figure 2:**
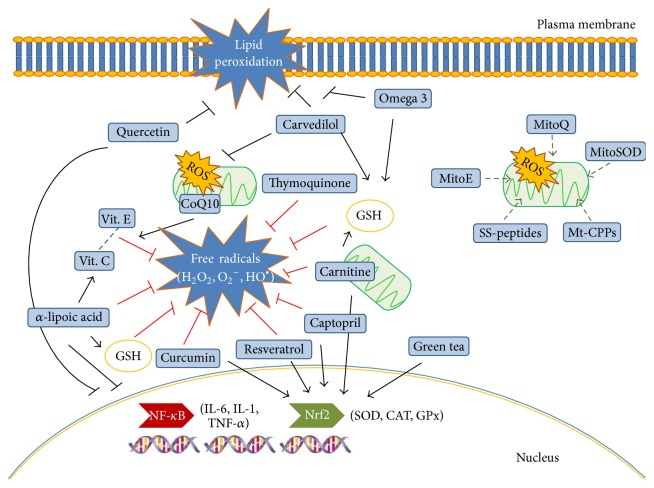
Site of action of most common endogenous and food derived antioxidants, phytochemicals, and conventional drugs with favorable antioxidant side effects and new available more selective anti-inflammatory medications. Some food derived antioxidants and drug (captopril) have both direct antioxidant effect acting as a scavenger of free radicals or inhibiting lipid peroxidation and indirect effect by modulating the activity of transcription factors NF-*κ*B and Nrf2. NF-*κ*B regulate the expression of proinflammatory genes (cytokines and chemokines) while Nrf2 mediates the synthesis of antioxidant enzymes such as SOD, CAT, and GPx. Carvedilol prevents mitochondrial dysfunction induced by oxidative stress and has protective effects against lipid peroxidation. GSH is a potent antioxidant depleted during oxidative stress and its level can be modulated by omega 3, carvedilol, L-carnitine, and alpha-lipoic acid. MitoQ, MitoSOD, mitoE, mt-CPPs, and SS-peptides are mitochondria-targeted molecules able to shuttle antioxidants into the mitochondria more efficiently than antioxidants molecules alone, in order to reduce ROS production.

A correct analysis of transcriptomic/microarray results may be useful to identify valuable biomarkers and to uncover new therapeutic targets for CKD. This strategy could also facilitate the employment of new available molecules/drugs in nephrology (Figure 2).

Mainly, endogenous and food derived antioxidants, phytochemicals, conventional drugs with favorable antioxidant side effects, and mitochondria-targeted molecules seem promising tools [9]. However, large randomized controlled clinical trials are still lacking.

Among endogenous and food derived antioxidants L-carnitine, coenzyme Q10 (CoQ10), alpha-lipoic acid (ALA), omega 3 polyunsaturated fatty acids (Omega-3 PUFAs), and vitamins E and vitamin C have demonstrated direct and indirect antioxidant actions in clinical studies conducted in CKD and HD patients [9].

Several phytochemicals such as thymoquinone (from* Nigella sativa* or black cumin), curcumin, quercetin, resveratrol, and green tea polyphenols are promising substances that could improve CKD outcome, acting against oxidative stress and preventing mitochondrial damage, as showed in numerous animal models. Clinical data are already available for curcumin supplementation in type-2 diabetic nephropathy, lupus nephritis, and CKD; it was able to reduce proteinuria and to prevent myocardial remodeling [9].

Also conventional and routinely used drugs may have favorable antioxidant side effects. For example, captopril has a thiol group in its structure that scavenges ROS and increases antioxidants enzyme levels.

Unfortunately, most of these molecules are unable to reach mitochondria at therapeutic dosage. For this reason “shuttle” molecules to better deliver antioxidants into the mitochondria are a growing field of interest.

First synthesized mitochondria target molecule was MitoE that is vitamin E conjugated with a lipophilic molecule, triphenylphosphonium (TPP). Then, with the same technique, MitoQ (a quinone linked to TPP), MitoSOD (a mimetic of MnSOD linked to TPP), and Mito-TEMPO (a nitroxide linked to TPP) were also produced. Phase I and II clinical trials are ongoing for MitoQ [9].

More recently even more efficacious peptides have been introduced: Szeto-Schiller peptides (SS) and mitochondrial cell-penetrating peptides (mt-CPPs) [127–129]. Two clinical trials are ongoing to test SS-31 efficacy to prevent ischemic-reperfusion injury in acute coronary events and to prevent renal function loss after renal artery angioplasty (NCT01755858).

Moreover, pharmaceutical companies are initiating new research programs to discover and develop potent, selective anti-inflammatory medications. Among them, bardoxolone methyl (activators of Nrf2) has been the first to reach full clinical development. It induces the antioxidant and cytoprotective transcription factor Nrf2, reduces the proinflammatory activity of the IKK-*β*/NF-*κ*B pathway, increases the production of antioxidant and reductive molecules, and decreases oxidative stress [130].

Another interesting compound is the primary amine of ethanolamine [131] (PEA), a naturally occurring N-acylethanolamine (NAE) that has protective effects in several animal models [132]. PEA acts locally penetrating the cells by passive transfer, due to its high lipophilicity, and by cells through a facilitated transport system [133].

PEA was first discovered in the late 1950s by studying the antiallergic and anti-inflammatory activity exerted by dietary supplementation with egg yolk, peanut oil, or soybean lecithin [134] due to a specific lipid fraction corresponding to PEA [135].

Anti-inflammatory and protective activities of PEA were confirmed in several models of inflammation, that is, carrageenan-induced paw edema, adjuvant-induced arthritis, tuberculin hypersensitivity, and ischemia reperfusion injury [131, 136, 137].

Finally, also peroxisome proliferator-activated receptors (PPARs), nuclear hormone receptors that stimulate transcription of genes by binding to specific DNA sequences, have demonstrated beneficial effects on vascular function [138]. In particular, PPAR-*α*, which is highly expressed in kidney, liver, and heart, has been shown to be involved in the control of blood pressure and consequently cardiovascular complications [139, 140].

Moreover PPAR-*α* agonists (fenofibrates) exert renoprotective effect through anti-inflammatory [141] and antioxidant properties via the downregulation of inflammatory cytokines and reduction of oxidative stress [142].

Moreover, the renoprotective effect of exogenous PPAR-*α* agonists (WY 14643 and ciprofibrate) through a regulation of megalin has been demonstrated in porcine epithelial cell line derived from kidney proximal tubule (LLC-PKI) and in BALB/c mice made albuminuric by bovine serum albumin (BSA) administration [143].

Therefore, to obtain the best results with these drugs it could be important to personalize administration and to properly identify the “right patient for the right medication.” Transcriptomic strategy could help to reach this objective.

## 6. Conclusion

Transcriptomic analysis, although still not largely employed in nephrology, has demonstrated great speculative potentialities. Several key regulators of the immune-inflammatory and oxidative stress pathways in CKD have been identified by using this innovative technology.

Additionally, it has demonstrated a unique capability to help clinicians to personalize patients' treatment according to their multigenetic expression fingerprint. A “customized” drug and diet administration (nutrigenomic and pharmacogenomic), by permitting the introduction of innovative therapeutic protocols including new antioxidant and anti-inflammatory compounds (e.g., endogenous and food derived antioxidants, phytochemicals, and mitochondria-targeted molecules), could definitely have a remarkable clinical and therapeutic impact.

Recently, M. R. Shahidi Bonjar and L. Shahidi Bonjar [144] have proposed and published an ideal and hypothetical device that, in future, could be used to improve homeostasis in patients with CKD. The device contains a quantitative microarray detector (QMD) which measures the concentrations of uremic waste and toxins of interest. These data are transmitted continuously to the homeostasis-oriented microarray column (HOMC) in order to communicate how much of each compound to retain. The patient's blood would flow through the tubing and proposed device to get close to constituents of the normal blood plasma near to homeostatic proportion. The device would be preprogrammed to remove set amounts of uremic waste and toxins from the blood, and the settings would be manually adjusted under the direction of a nephrologist according to the needs of the individual patient. When removal is sufficient, the QMD electronically detects the adequacy of hemodialysis or a near homeostasis condition and signals the end of treatment on the machine monitor.

However, to enforce the routine use of this methodology, researchers should work more to make data analysis and interpretation easily accessible to medical doctor not expert in statistics and bioinformatics and to reduce cost-consuming of microarray experiments.

For these reasons, at the moment, we are still far from large employment of this methodology in the nephrology research and in daily clinical practice. To achieve this objective a multidisciplinary network (including medical doctors, biologists, and statisticians) should be developed.

## Figures and Tables

**Table 1:** Relevant studies using transcriptomics in nephrology.

Reference	Comparison	Tissue/cells	Selected genes
[10]	HD versus PD versus CKD	PBMC	*Upregulated in HD*
			ATOX1, RELA, CSDE1, MIF, LTB4R, GSS, NFRKB
			*Upregulated in PD*
			HRH1, OLR1, CHST4, S100A8, CXCL12, GPX7
			*Upregulated in CKD*
			IL8RB, HDAC5, BCL6 P

[73]	PRE-HD versus POST-HD	Blood	*Upregulated POST-HD*
			TNF-A, IL-8, IL-18, IL-1RN, IL-4R, IL-10R, IFN-*γ*R1, CX3CR1, CXCR4, CCR7, C3aR1
	HD high CRP versus HD low CRP		*Upregulated in the low CRP group*
			IL-1RN, IL-4R, IL-10R, IFN-*γ*R1, CX3CR1, CXCR4, CCR7

[74]	HD versus HS	Muscle	*Upregulated in HD*
			SP3, MEF2A, MAF, TCF8, SMARCA1, DICER1, SFRS11, HMGN3, UPF3A, EPM2A, SOS2, DEK, CLK1, CDC10, LAF4, BMI1, DDX17, MAPK6, ANAPC13, MYBPC1, C6orf111, KIAA0740, ART3, BIRC2, RABGGTB, OA48-18, CSE1L, SH3GLB1, MAP2K4, GLRX, PIP5K3, SLC35A1, VPS26, PXMP1, SRP54, SCP-2, SUCLA2, DMD, PRDX3, NDUFA5, NRIP1, XPO1, PSMC6, SEPP1, AXOT, LANCL1, SHOC2, FAM8A1, UBE1C, UBL3, PJA2, YME1L1, ELF2, OGT, IRS1, GATM, DLD, BZRP, PICALM, CAST, ANGPT1, ANK3, AKAP9, Rif1, CBX3, CBX1, ZNF146, MYH8, Tl132, MORC3, ZC3H11A, PURA, FLJ13110, GBAS, KTN1, SLC30A9, Tre
			*Downregulated in HD*
			PTK9L, IGFBP4, TRAP1, TAX1BP3, LGALS3BP, GNAI2, HBA1, HBB
	PRE-HD versus POST-HD		*Upregulated in POST-HD*
			FST, GADD45A, GADD45B, IGFBP4, SAT, C-FOS, JUN-B, THBD, HES1, CCL2, CEBPD, BTG2, FOSL2, MYC, THBD, ZFP36, JE, NFIL3, SERPINB1, SCL39A14, NNMT, ARID5B
			*Downregulated in POST-HD*
			TOB1

[75]	semisynthetic versus full-synthetic dialysis membrane	PBMC	*Upregulated in HD using semisynthetic membranes* Immunity: ABCC5, ALOX12, CAMP, CCL2, CCR1, CD163, CLEC1B, CSF1R, CSF3R, CTSS, EPHX1, F2, FCGR1A, FGCR2B, GAB3, GBP1, GZMA, HLA-DQB1, HLA-DRB1, HLA-E, IRF7, ITGB1, KLRB1, LGALS9, LILRB3, LRRK2, LTB, MS4A2, PF4, PHCA, PPARA, PTPRC, RGS1, S100A8, SLA, TAP2, TGIF, TNFRSF8, XRCC6. Signal transduction: ABI3, ADRBK2, ARHGAP30, CCL2, CCR1, CENTA1, CHES1, CLEC1B, CSF1R, CSF3R, DOCK10, DTX4, ERBB3, FAS, FCGR1A, FCGR2B, FLJ23834, FPR1, GAB3, GABBR1, IFT140, ITGB1, LATS2, LILRA2, LILRB3, LIMS1, LRRK2, LTB, LTBP1, MAPK8, MS4A2, MYLK, NCF1, NRTN, P2RY13, PF4, PLCB2, PPARA, PTPN7, PTPRC, RAB9A, RASSF4, RGS1, RGS18, RGS6, SLA, SOCS3, TGIF, TNFRSF8, WSB2, WWTR1. Macrophage-mediated immunity: CD163, FCGR1A, FCGR2B, GAB3, GBP1, LTB, PF4, S100A8, TGIF. Natural killer cell-mediated immunity: CLEC1B, FCGR1A, FCGR2B, GZMA, KLRB1, LILRB3, LTB. T-cell-mediated immunity: CTSS, GAB3, GZMA, HLA-DQB1, HLA-DRB1, HLA-E, LTB, SLA, TNFRSF8. Cell motility: ABI3, CCR1, CSF1R, DOCK10, FPR1, ITGB1, LIMS1, LTBP1, PF4, S100A8, TUBB1. Cell surface receptor-mediated signal transduction: ADRBK2, CCL2, CCR1, CENTA1, CHES1, CSF1R, CSF3R, DTX4, ERBB3, FAS, FPR1, GAB3, GABBR1, IFT140, LIMS1, LTB, MS4A2, P2RY13, PF4, PLCB2, RGS1, RGS18, RGS6, SLA, TGIF, TNFRSF8. Apoptosis: FAS, GZMA, LGALS9, MYBL2, NAIP, NALP1, PDCD5, PF4, RASSF4, SOCS3, STK17B, TGIF. Intracellular protein traffic: CAMP, CD163, COMT, DOCK10, ERO1 L, IFT140, MX1, RAB9A, RASSF4, RIN1, RTN4, SSR2, STON2, STX5, TRAK2, TUBB1, YIPF5. Other metabolisms: AKR1C3, CA2, COMT, CRSP2, DIO2, GNPNAT1, MAP3K14, NEK2, PHCA, SLC27A3, SNAPC3. Cell cycle and cell cycle control: CCNB2, CYR61, EGFR, FOXF2, HTLF, JUN, KIF11, NBR2, TTK, TUBD1, UHRF1, WEE1

[76]	PMN stimulated with shredded hollow fibres of CU or PS versus unstimulated PMN	PMN	*Upregulated in PMN stimulated with shredded hollow fibres of CU or PS*
			AXUD1, FTH1, LIF, PTGS2, MGC12815, IL-1b, CCL3, CXCL1, SOCS3, PPIF, SPAG9, ACPP, DCT, GLA, GNS, PFKFB3, PLAU, USP36, SFRS3, DDX48, FLJ23231, PTD004, GNA13, HBEGF, DPYSL3, ARL8, GPR4, RASL11, DUSP2, EDN1, EDN3, EDNRB, JUN, FOS, EGR1, EGR2, DDIT3, EGR3, ELL2, NR4A3, TFAP2A, STAR, SEC31L1, ATP13A3, PHACTR1, TncRNA
			*Downregulated in PMN stimulated with shredded hollow fibres of CU or PS*
			FADD, FLI1, SOLH, YPEL3
	PMN stimulated with *E. Coli* versus unstimulated PMN		*Upregulated in PMN stimulated with E. Coli *
			GADD45B, BIRC3, IER3, IER5, SGK, CDH24, ICAM1, CSF1, VEGF, NBS1, CCL18, CCL20, CCL3, CD48, CXCL1, CXCL2, CXCL3, IL1A, IL1B, IL1RN, LIF, MGC12815, NFIL3, PTGS2, SOCS3, TNF, TNFAIP6, EGR1, EGR2, ETS2, HIVEP1, ISL1, JUN, MAFF, MAFG, NFKB1, NFKBIA, NFKBIE, NFKBIZ, NR4A3, TFAP2A, TNFAIP3, XBP1, ZFHX1B, B4GALT5, DCT, FPGS, GCH1, GLA, GNPDA1, LOC285533, OAZIN, PLAU, PPIF, PPP1R15B, DDX48, FLJ23231, NMES1, SFMBT2, SNAPC3, TIFA, PHACTR1, ARL8, CALCA, CDC42EP3, DPYSL3, DUSP2, EDN1, EDN3, EHD1, GAB2, GPR4, MAPK6, NSMAF, SLC35B2, RHCG, SPAG9, VANGL1, VPS18, KCNJ2, AQP9
			*Downregulated in PMN stimulated with E. Coli *
			FADD, LAMB1, MEF2C, HNRPUL1, NDP52, YPEL3, DUSP6
	PMN stimulated with *E. Coli* versus PMN stimulated with HD membrane		*Upregulated in PMN stimulated with E. Coli *
			CCL20, CXCL3, CCL3, IL1A, TNF, NFKBIA, NFKBIE, NFKBIZ, NFKB1, TNFAIP3, PLAU, IER5, ICAM3

[29]	HD versus CKD III-IV	PBMC	*Upregulated in HD* AIF1, ATRN, BCL2, C1QBP, CADM1, CCL3, CD163, CD1D, CD300C, CD4, CD58, CD83, CD86, CEBPG, CLEC2B, CLEC4A, CLEC5A, CNIH, CTSC, CTSS, CXCL2, CXCL3, DNAJC8, EREG, FCAR, FTH1, FUS, GPR183, GPR65, GZMA, HLA-DMA, HLA-DMB, HLA-DPA1, HLA-DQB1, HLA-DRA, HLA-DRB1, ICOS, IFI30, IFI44, IGBP1, IL15, IL6ST, IL8, ILF2, ITGB1, ITGB2, KLRB1, KLRC1, KLRC3, KLRD1, KLRF1, LILRB2, LY86, LY96, NFIL3, PLA2G7, POMP, PRKRA, PTGER4, PTX3, RGS1, SAMHD1, SH2D1A, SIK1, SPN, TNFRSF25, TNFSF13, TNFSF8, VIPR1, XCL1, YTHDF2 *Downregulated in HD* ACSL1, AIM2, ALOX15, ALOX5AP, ANXA11, APOL2, AQP9, ARHGDIB, ATP6V0A2, B2M, BCL2, C4A, CD24, CD27, CD2BP2, CD40, CD40LG, CD53, CD59, CD70, CD80, CD8B, CD97, CEACAM8, CNOT1, CNR2, DCLRE1C, DPP8, ELF4, FAIM3, FASLG, FCGR2A, FCGR2C, FCGR3A, FCGR3B, FYB, GBP1, GEM, GPR44, GPR68, GPSM3, GTF2I, GTPBP1, HLA-A, HLA-B, HLA-C, HLA-DOB, HLA-DPA1, HLA-DRB1, HLA-E, HLA-F, HLA-G, IFI16, IFI44, IFIT2, IFIT3, IFITM1, IFITM2, IFITM3, IGHA1, IGHD, IGHG1, IGKC, IGLL3P, IKBKG, IL16, IL18RAP, IL1A, IL1R1, IL1R2, IL1RAP, IL1RN, IL2RG, IL6R, IL7R, ILT4, IRF1, IRF2, IRF9, KIR2DL1, KLRG1, KRT1, LAT, LAT2, LILRA2, LILRB3, LSP1, LST1, LTF, LY9, LYST, MADCAM1, MAPRE2, MASP2, MBP, MNDA, MR1, MX2, MYD88, NCF2, NCF4, NCR2, NOTCH1, OASL, OTUB1, PGLYRP1, PLUNC, PSMB9, PSME1, PTAFR, PYHIN1, REG3A, S100A9, S1PR4, SECTM1, SEMA4D, SERPINA1, SIT1, SLC11A1, SLC4A1, ST6GAL1, TAPBP, TARP, TGFB2, TLR2, TLR6, TNFAIP6, TNFRSF13B, TNFRSF25, TNFRSF9, TNFSF10, TNFSF14, TOLLIP, TRAC, TRBC1, TRBC2, TREM1, TYROBP

[77]	HD versus HS	Blood	*Upregulated in HD* MORN1, FGF18, ZNF205, ADARB1, GLTSCR2, SAP30L, ODF3B, SRCRB4D, TNPO2, MAPRE3, DUX4, RUNX3, PCGF5, GPR144, MAPK8IP2, ZFPL1, PLEKHG5, ELAVL3, FBXO44, UBE2J2, IL34, SLC25A37, SFTPC, PDLIM7, ARMC5, PLEKHN1, GNAS, EPN1, BCL2, CHCHD5, TFRC, RPS11, LMNA, CYP11B2, TMEFF2, GP1BB, TRIM8, VRTN *Downregulated in HD* ATP2A3, MESDC1, FBRSL1, RNF19B, ATPIF1, FKBP1A, ILF3, RBBP4, PEBP1, CTBP1, HINT1, KLHL24, KDM1B, MTA1, KCTD5, CCDC115, SLC23A2, ACAD8, RAB11FIP4, RNF19B, NONO, TNRC6A, NDUFB8, OGT, ATP5C1, MARCH5, PPP1R8, RALGAPB, IRF2, ESYT2, BHLHE40, RABGAP1, GABPB2, QKI, FLI1, RAB7A, PDCD4, BCL9L, RNF166, ACTL6A, S1PR1, GLUD1, CCDC88C, CSAD, ADSS, SRSF1, LOC93622, ACLY, PRF1, MAPK9, KLF7, PIK3IP1, TRIB2

[78]	Mice with intraperitoneal injection of chlorhexidine gluconate versus control	Parietal peritoneum	*Upregulated in parietal peritoneum treated with chlorhexidine gluconate* PTGS2, COL8a1, Ddx3y, EIF2S3Y, ADAM12, Il6, CXCL1, MMP14, CCL7, ANKRD1, LRRC15, CCL2, G1p2, RUNX1, IRF7, CTHRC1, CYP7B1, PTN, COL5A2, FN1, CSPG2, THBS1, MS4A4C, LOX, GDF15, DNM3OS, RIAN, WISP1, SFRP1, COL3A1, STAT2, OASL2, TNC, NCAM1, ITGA5, TRIB3, GJA1, IFIT2, SERPINA3N, LOXL2, GLIPR2, OASL1, TIMP1 *Downregulated in parietal peritoneum treated with chlorhexidine gluconate* IGH4

[79]	PD/HD versus CKD III-IV/HS	PBMC	*Upregulated in HD/PD* B4GALT1, ANG, B3GNT2, CHPF2, CHST11, CHST2, CHSY1, CSGALNACT2, DSE, ERBB2IP, GNS, GUSB, HBEGF, HEXA, HEXB, HPSE, HS2ST1, RPL29, SGSH, SMC3, SOD1, TGFB1, VCAN, VEGFA, XYLT1 *Downregulated in HD/PD* COL4A3, ACAN, AGRN, B3GNT1, B4GALT1, B4GALT3, B4GALT7, CHI3L1, COL7A1, CHPF, CHST1, B4GALT7, CHST15, CHST3, CHST8, COL13A1, COL18A1, COL1A2, COL5A3, COL9A2, DMD, DST, EXTL3, FBLN1, FMOD, GALNS, HABP2, HDGF, HEXA, IDS, LAMA2, LTBP2, NID2, LTBP4, MATN1, NAGLU, NDST1, NID2, PI3, PRSS1, SGCD, SNTB2, ST3GAL2, TGFB1, THBS4, TNFAIP6

[9]	HD versus CKD II-III versus HS	PBMC	*Upregulated in HD* LSM3, TNFAIP3, JUNB, SFRS10, KLF4, MBD2, RPL36A, CLEC2B, CDV3, HMGN3, DNTTIP2, H3F3B, ZC3H15, TAF9, UQCRB, SNRPG, DBI, NDUFB1, UQCRH, COX6C, EEF1B2, NDUFA6, LOC732102, NDUFS5, HMGB1, DBI, TINP1, ATP5I, ATP5O, PFDN5, ATP5J, RPL23, RPS7, RPL34, TAX1BP1, PSMA6, SNRPE, HSP90AA1, ATG5, NDUFA1, AIF1, LSM7, NPM1, COX7C

[80]	HD versus HS	PBMC	*Upregulated in HD* CASP1, NLRP3, NAIP5 *Downregulated in HD* NLRP1, TXNIP, CARD8,

## Abbreviations

CKD: Chronic kidney disease
GFR: Glomerular filtration rate
ESRD: End stage renal disease
RRT: Renal replacement therapies
HD: Hemodialysis
PD: Peritoneal dialysis
ACE: Angiotensin-converting enzyme
ARBs: Angiotensin receptor blockers
PBMCs: Peripheral blood mononuclear cells
TNF-*α*: Tumor necrosis factor-*α*
PTX3: Pentraxin-3
TWEAK: TNF-like weak inducer of apoptosis
ROS: Reactive oxygen species
OXPHOS: Oxidative phosphorylation system
NADH: Nicotinamide adenine dinucleotide
FADH_2_: Flavin adenine dinucleotide
mtDNA: Mitochondrial DNA
O_2_^−^: Superoxide anion
CRP: C-reactive protein
HS-CRP: High-sensitive C reactive protein
PMN: Polymorphonuclear neutrophils
HLA: Human leukocyte antigen
NK: Natural killer
CG: Chlorhexidine gluconate
HS: Healthy subjects
HPSE: Heparanase
PAMPs: Pathogen-associated molecular patterns
DAMPs: Damage-associated molecular patterns
ALA: Alpha-lipoic acid
CoQ10: Coenzyme Q10
Omega 3 PUFA: Omega 3 polyunsaturated fatty acids
TPP: Triphenylphosphonium
SS: Szeto-Schiller
Mt-CPPs: Mitochondrial cell-penetrating peptides
Nrf2: Nuclear factor erythroid 2-related factor 2
PEA: Primary amine ethanolamine
NAE: N-Acylethanolamine
PPARs: Peroxisome proliferator-activated receptors
BSA: Bovine serum albumin
QMD: Quantitative microarray detector
HOMC: Homeostasis-oriented microarray column.
